# 6-[(2-Methyl­phen­yl)sulfan­yl]-5-propyl­pyrimidine-2,4(1*H*,3*H*)-dione

**DOI:** 10.1107/S1600536814013269

**Published:** 2014-06-14

**Authors:** Nadia G. Haress, Hazem A. Ghabbour, Ali A. El-Emam, C. S. Chidan Kumar, Hoong-Kun Fun

**Affiliations:** a Department of Pharmaceutical Chemistry, College of Pharmacy, King Saud University, PO Box 2457, Riaydh 11451, Saudi Arabia; b King Abdullah Institute for Nanotechnology (KAIN), King Saud University, Riyadh 11451, Saudi Arabia; c X-ray Crystallography Unit, School of Physics, Universiti Sains Malaysia, 11800 USM, Penang, Malaysia

## Abstract

In the title pyrimidine-2,4-dione derivative, C_14_H_16_N_2_O_2_S, the dihedral angle between the six-membered rings is 77.81 (10)°. The mol­ecule is twisted about the C_p_—S (p = pyrimidine) bond, with a C—S—C—N torsion angle of −59.01 (17)°. An intramolecular C—H⋯S hydrogen bond generates an *S*(5) ring motif. In the crystal, bifurcated acceptor N—H⋯O and C—H⋯O hydrogen bonds generate inversion-related dimers incorporating *R*_2_^1^(9) and *R*_2_^2^(8) loops. These dimers are connected into a chain extending along the *a*-axis direction by a second pair of inversion-related N—H⋯O hydrogen bonds, forming another *R*_2_^2^(8) loop. The crystal structure is further stabilized by weak inter­molecular C—H⋯π inter­actions, generating a three-dimensional network.

## Related literature   

For the pharmacological activity of pyrimidine-2,4-dione derivatives, see: Al-Abdullah *et al.* (2011 ▶, 2014 ▶); Tanaka *et al.* (1995 ▶); Hopkins *et al.* (1996 ▶); Russ *et al.* (2003 ▶); Al-Deeb *et al.* (2013 ▶); Nencka *et al.* (2006 ▶); El-Emam *et al.* (2004 ▶); El-Brollosy *et al.* (2009 ▶, 2011 ▶). For related pyrimidine-2,4-dione structures, see: Al-Omary *et al.* (2014 ▶); Wang *et al.* (2006 ▶). For reference bond lengths, see: Allen *et al.* (1987 ▶). For hydrogen-bond motifs, see: Bernstein *et al.* (1995 ▶).
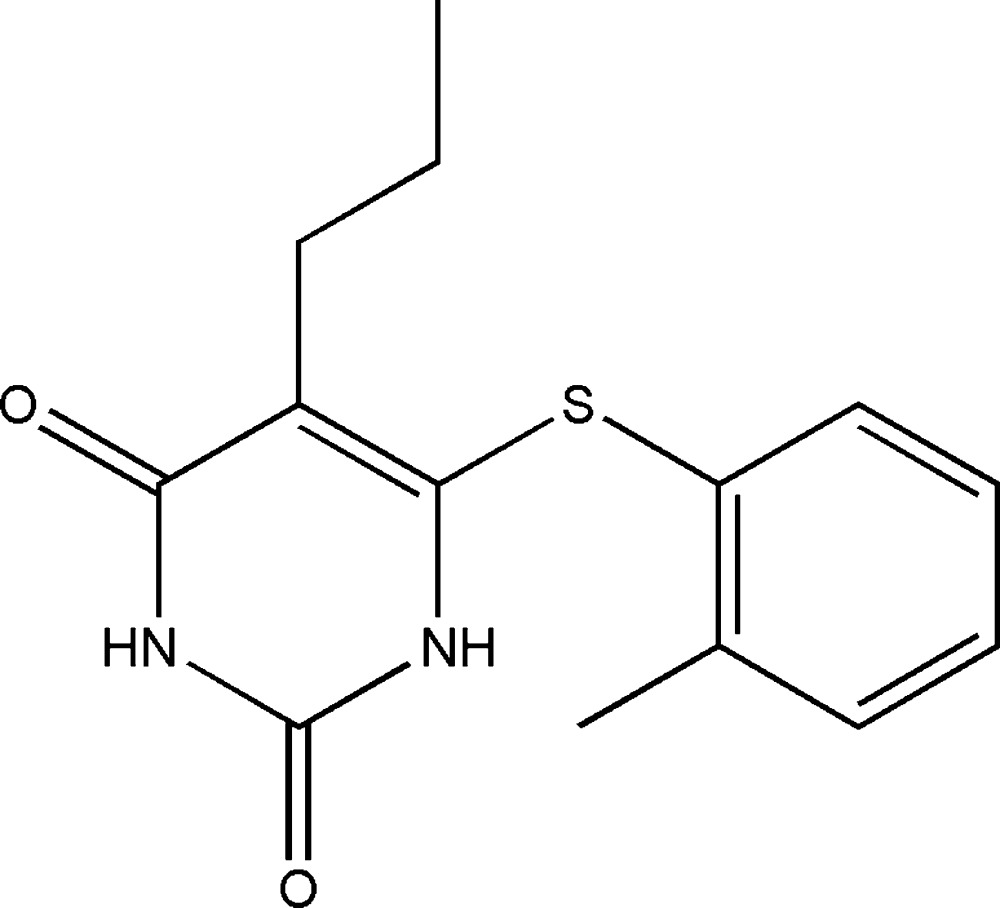


## Experimental   

### 

#### Crystal data  

C_14_H_16_N_2_O_2_S
*M* *_r_* = 276.36
Monoclinic, 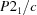

*a* = 10.3434 (8) Å
*b* = 5.3355 (3) Å
*c* = 24.4948 (18) Å
β = 91.171 (3)°
*V* = 1351.52 (16) Å^3^
*Z* = 4
Mo *K*α radiation
μ = 0.24 mm^−1^
*T* = 293 K
0.42 × 0.11 × 0.06 mm

#### Data collection  

Bruker APEXII CCD diffractometer
Absorption correction: multi-scan (*SADABS*; Bruker, 2009 ▶) *T*_min_ = 0.906, *T*_max_ = 0.986
32195 measured reflections
4165 independent reflections
2968 reflections with *I* > 2σ(*I*)
*R*_int_ = 0.088

#### Refinement  

*R*[*F* ^2^ > 2σ(*F* ^2^)] = 0.063
*wR*(*F* ^2^) = 0.134
*S* = 1.08
4165 reflections
182 parameters
H atoms treated by a mixture of independent and constrained refinement
Δρ_max_ = 0.56 e Å^−3^
Δρ_min_ = −0.37 e Å^−3^

### 

Data collection: *APEX2* (Bruker, 2009 ▶); cell refinement: *SAINT* (Bruker, 2009 ▶); data reduction: *SAINT*; program(s) used to solve structure: *SHELXS97* (Sheldrick, 2008 ▶); program(s) used to refine structure: *SHELXL97* (Sheldrick, 2008 ▶); molecular graphics: *SHELXTL* (Sheldrick, 2008 ▶); software used to prepare material for publication: *SHELXTL* and *PLATON* (Spek, 2009 ▶).

## Supplementary Material

Crystal structure: contains datablock(s) global, I. DOI: 10.1107/S1600536814013269/sj5409sup1.cif

Structure factors: contains datablock(s) I. DOI: 10.1107/S1600536814013269/sj5409Isup2.hkl

Supporting information file. DOI: 10.1107/S1600536814013269/sj5409Isup3.cml

CCDC reference: 1007120

Additional supporting information:  crystallographic information; 3D view; checkCIF report

## Figures and Tables

**Table 1:** Hydrogen-bond geometry (Å, °) *Cg*1 and *Cg*2 are the centroids of C1–C6 and C8–C11/N1/N2 rings, respectively.

*D*—H⋯*A*	*D*—H	H⋯*A*	*D*⋯*A*	*D*—H⋯*A*
C12—H12*B*⋯S1	0.97	2.75	3.166 (2)	107
N2—H1*N*2⋯O2^i^	0.82 (2)	2.01 (2)	2.829 (2)	171 (2)
N1—H1*N*1⋯O1^ii^	0.83 (3)	1.98 (3)	2.805 (2)	173 (2)
C7—H7*B*⋯O1^ii^	0.96	2.58	3.289 (3)	131
C2—H2*A*⋯*Cg*2^iii^	0.93	2.91	3.700 (2)	144
C7—H7*B*⋯*Cg*1^iv^	0.96	2.85	3.632 (3)	140

Symmetry codes: (i) 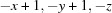; (ii) 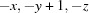; (iii) 
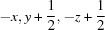
; (iv) 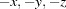.

**Fig. 1.**
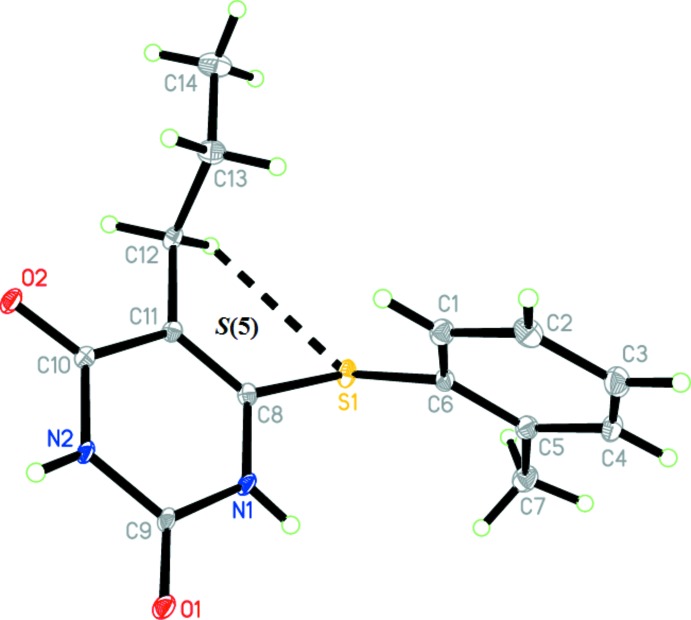
The molecular structure of the title compound with atom labels and 30% probability displacement ellipsoids.

**Fig. 2.**
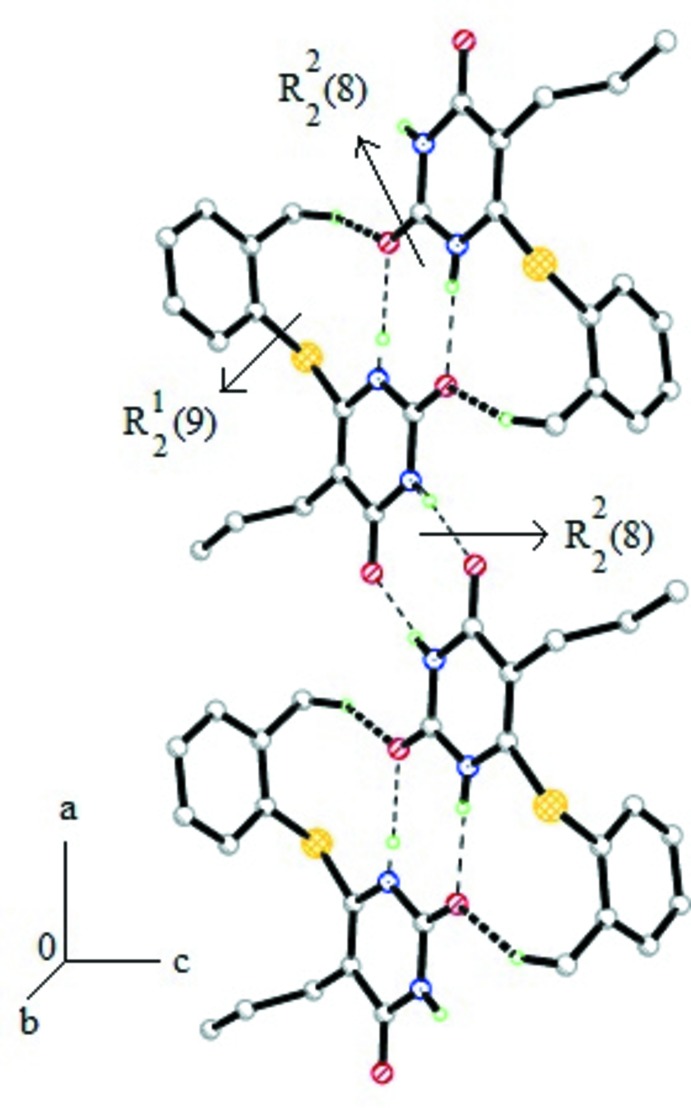
Crystal packing of the title compound, showing the hydrogen bonding interactions as dashed lines. H-atoms not involved in the hydrogen bonding are omited for clarity.

## supplementary crystallographic information

### S1. Comment

Pyrimidine-2,4-diones and their related derivatives have long been known for their diverse chemotherapeutic activities (Al-Abdullah *et al.*, 2014, Al-Deeb *et al.*, 2013) including antiviral activity against HIV (Tanaka *et al.*, 1995; Hopkins *et al.*, 1996; El-Emam *et al.*, 2004), and HSV viruses (Russ *et al.*, 2003). In addition, potent anticancer activity was observed for several pyrimidine-2,4-diones (Nencka *et al.*, 2006). In a continuation of our interest in the chemical and pharmacological properties of pyrimidine and uracil derivatives (Al-Abdullah *et al.*, 2011; El-Brollosy *et al.*, 2009), we have synthesized the title compound (I) as a potential chemotherapeutic agent.

In the title compound (Fig. 1), the two six-membered rings (C1–C6 and C8–C11/N1/N2) are essentially planar, with maximum deviations of -0.012 (2) Å at atom C5 and 0.020 (2) Å at atom C10, respectively. The molecule is bent at the S atom with C6–S1–C8–N1 torsion angle of -59.01 (17)°. The heterocycle containing the structural unit CON_2_H_2_CO forms a dihedral of 77.81 (10)° with the adjacent benzene ring. Bond lengths and angles in (I) show normal values (Allen *et al.*, 1987) and are comparable with those in related structures (Al-Omary *et al.*, 2014; El-Brollosy *et al.*, 2011; Wang *et al.*, 2006). An intramolecular C—H···S hydrogen bond generates an *S*(5) ring motif. In the crystal structure, bifurcated acceptor N1–H1N1···O1 and C7–H7B···O1 (Table 1) hydrogen bonds link the two adjacent molecules into centrosymmetric inversion related dimers incorparating *R*_2_^1^(9) and *R*_2_^2^(8) loops (Fig. 2, Bernstein *et al.*, 1995). These dimers are connected into a chain extending along *a*-axis direction *via* a pair of N2–H1N2···O2 hydrogen bonds (Table 1) resulting in another *R*_2_^2^(8) loop (Fig. 2, Bernstein *et al.*, 1995). The crystal structure stability is further consolidated by weak intermolecular C–H···π interactions (Table 1) involving the centroids of the six-membered C8–C11/N1/N2 (*C*g1) and C1–C6 benzene (*C*g2) rings.

### S2. Experimental

A mixture of 6-chloro-5-propyluracil (943 mg, 0.005 mol), *o*-thiocresol (621 mg, 0.005 mol) and potassium hydroxide (281 mg, 0.005 mol), in ethanol (10 ml), was heated under reflux for 3 h. The solvent was then distilled off in vaccuo and the residue was washed with cold water, dried and crystallized from ethanol to yield 940 mg (68%) of the title compound (C_14_H_16_N_2_O_2_S) as colorless needle crystals. M·P.: 210–212 °C.

^1^H NMR (DMSO-d_6_, 500.13 MHz): δ 0.84 (t, 3H, CH_2_CH_3_, J = 7.0 Hz), 1.37–1.40 (m, 2H, CH_2_CH_3_), 2.33 (s, 3H, Ar—CH_3_), 2.43 (t, 2H, CH_2_CH_2_CH_3_, J = 7.0 Hz), 6.92–7.02 (m, 3H, Ar—H), 7.26–7.28 (m, 1H, Ar—H), 10.91 (s, 1H, NH), 11.24 (s, 1H, NH). 13 C NMR (DMSO-d_6_, 125.76 MHz): δ 13.72 (CH_2_CH_3_), 22.06 (CH_2_CH_3_), 20.12 (Ar—CH_3_), 28.22 (CH_2_CH_2_CH_3_), 117.44 (Pyrimidine C-5), 125.90, 126.50, 129.88, 130.20, 133.18, 140.56 (Ar—C), 143.02 (Pyrimidine C-6), 150.53 (C=O), 163.23 (C=O).

### S3. Refinement

The nitrogen-bound H-atoms were located in a difference Fourier map and were refined freely. Other H atoms were positioned geometrically (C=H 0.93–0.97 Å) and refined using a riding model with *U*_iso_(H) = 1.2 *U*_eq_(C) or 1.5 *U*_eq_(C) for methyl H atoms. A rotating group model was used for the methyl group.

### Crystal data

**Table:**

C_14_H_16_N_2_O_2_S	*F*(000) = 584
*M**_r_* = 276.36	*D*_x_ = 1.353 Mg m^−^^3^
Monoclinic, *P*2_1_/*c*	Mo *K*α radiation, λ = 0.71073 Å
Hall symbol: -P 2ybc	Cell parameters from 7715 reflections
*a* = 10.3434 (8) Å	θ = 2.6–30.3°
*b* = 5.3355 (3) Å	µ = 0.24 mm^−^^1^
*c* = 24.4948 (18) Å	*T* = 293 K
β = 91.171 (3)°	Plate, colourless
*V* = 1351.52 (16) Å^3^	0.42 × 0.11 × 0.06 mm
*Z* = 4	

### Data collection

**Table:**

Bruker APEXII CCD diffractometer	4165 independent reflections
Radiation source: fine-focus sealed tube	2968 reflections with *I* > 2σ(*I*)
Graphite monochromator	*R*_int_ = 0.088
φ and ω scans	θ_max_ = 30.7°, θ_min_ = 2.6°
Absorption correction: multi-scan (*SADABS*; Bruker, 2009)	*h* = −14→14
*T*_min_ = 0.906, *T*_max_ = 0.986	*k* = −7→7
32195 measured reflections	*l* = −34→35

### Refinement

**Table:**

Refinement on *F*^2^	Primary atom site location: structure-invariant direct methods
Least-squares matrix: full	Secondary atom site location: difference Fourier map
*R*[*F*^2^ > 2σ(*F*^2^)] = 0.063	Hydrogen site location: inferred from neighbouring sites
*wR*(*F*^2^) = 0.134	H atoms treated by a mixture of independent and constrained refinement
*S* = 1.08	*w* = 1/[σ^2^(*F*_o_^2^) + (0.0398*P*)^2^ + 1.8642*P*] where *P* = (*F*_o_^2^ + 2*F*_c_^2^)/3
4165 reflections	(Δ/σ)_max_ < 0.001
182 parameters	Δρ_max_ = 0.56 e Å^−^^3^
0 restraints	Δρ_min_ = −0.37 e Å^−^^3^

### Special details

**Table:**

Geometry. All e.s.d.'s (except the e.s.d. in the dihedral angle between two l.s. planes) are estimated using the full covariance matrix. The cell e.s.d.'s are taken into account individually in the estimation of e.s.d.'s in distances, angles and torsion angles; correlations between e.s.d.'s in cell parameters are only used when they are defined by crystal symmetry. An approximate (isotropic) treatment of cell e.s.d.'s is used for estimating e.s.d.'s involving l.s. planes.
Refinement. Refinement of *F*^2^ against ALL reflections. The weighted *R*-factor *wR* and goodness of fit *S* are based on *F*^2^, conventional *R*-factors *R* are based on *F*, with *F* set to zero for negative *F*^2^. The threshold expression of *F*^2^ > σ(*F*^2^) is used only for calculating *R*-factors(gt) *etc*. and is not relevant to the choice of reflections for refinement. *R*-factors based on *F*^2^ are statistically about twice as large as those based on *F*, and *R*- factors based on ALL data will be even larger.

### Fractional atomic coordinates and isotropic or equivalent isotropic displacement parameters (Å^2^)

**Table:**

	*x*	*y*	*z*	*U*_iso_*/*U*_eq_	
S1	0.06364 (5)	−0.09820 (10)	0.09629 (2)	0.01784 (14)	
O1	0.14557 (13)	0.6290 (3)	−0.02258 (6)	0.0174 (3)	
O2	0.50699 (13)	0.2361 (3)	0.04241 (6)	0.0185 (3)	
N1	0.12191 (17)	0.2935 (3)	0.03381 (7)	0.0144 (4)	
N2	0.32504 (16)	0.4210 (4)	0.00837 (7)	0.0141 (4)	
C1	0.0464 (2)	0.3038 (4)	0.16726 (9)	0.0199 (5)	
H1A	0.1358	0.3149	0.1645	0.024*	
C2	−0.0179 (2)	0.4673 (5)	0.20078 (9)	0.0239 (5)	
H2A	0.0274	0.5909	0.2199	0.029*	
C3	−0.1512 (2)	0.4459 (5)	0.20570 (10)	0.0262 (5)	
H3A	−0.1952	0.5532	0.2288	0.031*	
C4	−0.2185 (2)	0.2647 (5)	0.17622 (10)	0.0237 (5)	
H4A	−0.3075	0.2514	0.1801	0.028*	
C5	−0.1558 (2)	0.1012 (4)	0.14083 (9)	0.0195 (4)	
C6	−0.0214 (2)	0.1221 (4)	0.13752 (8)	0.0163 (4)	
C7	−0.2318 (2)	−0.0789 (5)	0.10612 (10)	0.0247 (5)	
H7A	−0.2013	−0.2463	0.1128	0.037*	
H7B	−0.2214	−0.0378	0.0683	0.037*	
H7C	−0.3217	−0.0684	0.1150	0.037*	
C8	0.17527 (19)	0.1011 (4)	0.06450 (8)	0.0139 (4)	
C9	0.19324 (18)	0.4591 (4)	0.00465 (8)	0.0136 (4)	
C10	0.38780 (19)	0.2403 (4)	0.03944 (8)	0.0140 (4)	
C11	0.30560 (19)	0.0645 (4)	0.06833 (8)	0.0136 (4)	
C12	0.3691 (2)	−0.1357 (4)	0.10241 (8)	0.0154 (4)	
H12A	0.4479	−0.1886	0.0849	0.018*	
H12B	0.3119	−0.2795	0.1039	0.018*	
C13	0.4024 (2)	−0.0509 (4)	0.16085 (9)	0.0220 (5)	
H13A	0.4635	0.0867	0.1597	0.026*	
H13B	0.3246	0.0095	0.1780	0.026*	
C14	0.4602 (3)	−0.2630 (5)	0.19489 (10)	0.0304 (6)	
H14A	0.4831	−0.2017	0.2306	0.046*	
H14B	0.5362	−0.3257	0.1776	0.046*	
H14C	0.3980	−0.3955	0.1979	0.046*	
H1N2	0.367 (2)	0.530 (5)	−0.0072 (10)	0.017 (6)*	
H1N1	0.042 (3)	0.304 (5)	0.0290 (10)	0.024 (7)*	

### Atomic displacement parameters (Å^2^)

**Table:**

	*U*^11^	*U*^22^	*U*^33^	*U*^12^	*U*^13^	*U*^23^
S1	0.0144 (2)	0.0136 (3)	0.0258 (3)	−0.0032 (2)	0.00659 (19)	0.0002 (2)
O1	0.0098 (6)	0.0184 (8)	0.0241 (8)	−0.0012 (6)	0.0003 (6)	0.0051 (6)
O2	0.0089 (7)	0.0201 (8)	0.0264 (8)	−0.0011 (6)	0.0001 (6)	0.0052 (7)
N1	0.0064 (8)	0.0165 (9)	0.0202 (9)	−0.0015 (7)	0.0003 (7)	0.0019 (7)
N2	0.0070 (7)	0.0167 (9)	0.0185 (9)	−0.0019 (7)	0.0016 (6)	0.0028 (7)
C1	0.0178 (10)	0.0194 (11)	0.0226 (11)	−0.0012 (9)	0.0027 (9)	0.0026 (9)
C2	0.0301 (12)	0.0210 (12)	0.0207 (11)	−0.0014 (10)	0.0017 (10)	0.0004 (9)
C3	0.0282 (12)	0.0257 (13)	0.0249 (12)	0.0081 (10)	0.0072 (10)	0.0019 (10)
C4	0.0175 (10)	0.0287 (13)	0.0251 (12)	0.0053 (9)	0.0058 (9)	0.0032 (10)
C5	0.0165 (10)	0.0203 (11)	0.0219 (11)	−0.0011 (9)	0.0027 (8)	0.0051 (9)
C6	0.0146 (9)	0.0177 (10)	0.0166 (10)	0.0011 (8)	0.0034 (8)	0.0045 (8)
C7	0.0194 (11)	0.0250 (12)	0.0298 (12)	−0.0024 (10)	0.0006 (9)	0.0016 (10)
C8	0.0131 (9)	0.0126 (9)	0.0160 (9)	−0.0038 (8)	0.0015 (7)	−0.0006 (8)
C9	0.0077 (8)	0.0175 (10)	0.0156 (10)	−0.0031 (7)	0.0003 (7)	−0.0026 (8)
C10	0.0121 (9)	0.0143 (10)	0.0157 (10)	0.0004 (8)	−0.0001 (8)	−0.0013 (8)
C11	0.0127 (9)	0.0131 (10)	0.0150 (9)	−0.0019 (8)	0.0001 (7)	−0.0015 (8)
C12	0.0120 (9)	0.0131 (10)	0.0211 (10)	−0.0004 (8)	0.0010 (8)	−0.0004 (8)
C13	0.0250 (11)	0.0189 (12)	0.0221 (11)	0.0005 (9)	−0.0022 (9)	0.0010 (9)
C14	0.0389 (15)	0.0267 (13)	0.0253 (13)	0.0045 (11)	−0.0075 (11)	0.0032 (10)

### Geometric parameters (Å, º)

**Table:**

S1—C8	1.763 (2)	C4—H4A	0.9300
S1—C6	1.792 (2)	C5—C6	1.399 (3)
O1—C9	1.223 (3)	C5—C7	1.496 (3)
O2—C10	1.234 (2)	C7—H7A	0.9600
N1—C9	1.362 (3)	C7—H7B	0.9600
N1—C8	1.381 (3)	C7—H7C	0.9600
N1—H1N1	0.83 (3)	C8—C11	1.363 (3)
N2—C9	1.380 (2)	C10—C11	1.459 (3)
N2—C10	1.382 (3)	C11—C12	1.499 (3)
N2—H1N2	0.82 (3)	C12—C13	1.534 (3)
C1—C2	1.379 (3)	C12—H12A	0.9700
C1—C6	1.394 (3)	C12—H12B	0.9700
C1—H1A	0.9300	C13—C14	1.521 (3)
C2—C3	1.391 (3)	C13—H13A	0.9700
C2—H2A	0.9300	C13—H13B	0.9700
C3—C4	1.386 (4)	C14—H14A	0.9600
C3—H3A	0.9300	C14—H14B	0.9600
C4—C5	1.398 (3)	C14—H14C	0.9600
			
C8—S1—C6	100.76 (10)	C11—C8—N1	121.85 (18)
C9—N1—C8	123.55 (17)	C11—C8—S1	122.60 (16)
C9—N1—H1N1	115.4 (19)	N1—C8—S1	115.52 (14)
C8—N1—H1N1	120.6 (19)	O1—C9—N1	123.35 (18)
C9—N2—C10	126.26 (18)	O1—C9—N2	122.14 (18)
C9—N2—H1N2	112.9 (17)	N1—C9—N2	114.51 (18)
C10—N2—H1N2	120.4 (17)	O2—C10—N2	120.18 (19)
C2—C1—C6	120.5 (2)	O2—C10—C11	123.46 (19)
C2—C1—H1A	119.7	N2—C10—C11	116.35 (17)
C6—C1—H1A	119.7	C8—C11—C10	117.37 (19)
C1—C2—C3	119.4 (2)	C8—C11—C12	124.19 (18)
C1—C2—H2A	120.3	C10—C11—C12	118.36 (17)
C3—C2—H2A	120.3	C11—C12—C13	113.39 (18)
C4—C3—C2	120.0 (2)	C11—C12—H12A	108.9
C4—C3—H3A	120.0	C13—C12—H12A	108.9
C2—C3—H3A	120.0	C11—C12—H12B	108.9
C3—C4—C5	121.6 (2)	C13—C12—H12B	108.9
C3—C4—H4A	119.2	H12A—C12—H12B	107.7
C5—C4—H4A	119.2	C14—C13—C12	111.74 (19)
C4—C5—C6	117.4 (2)	C14—C13—H13A	109.3
C4—C5—C7	120.6 (2)	C12—C13—H13A	109.3
C6—C5—C7	122.0 (2)	C14—C13—H13B	109.3
C1—C6—C5	121.0 (2)	C12—C13—H13B	109.3
C1—C6—S1	120.23 (16)	H13A—C13—H13B	107.9
C5—C6—S1	118.71 (17)	C13—C14—H14A	109.5
C5—C7—H7A	109.5	C13—C14—H14B	109.5
C5—C7—H7B	109.5	H14A—C14—H14B	109.5
H7A—C7—H7B	109.5	C13—C14—H14C	109.5
C5—C7—H7C	109.5	H14A—C14—H14C	109.5
H7A—C7—H7C	109.5	H14B—C14—H14C	109.5
H7B—C7—H7C	109.5		
			
C6—C1—C2—C3	1.5 (3)	C8—N1—C9—O1	179.84 (19)
C1—C2—C3—C4	−1.3 (4)	C8—N1—C9—N2	−0.4 (3)
C2—C3—C4—C5	−0.5 (4)	C10—N2—C9—O1	177.7 (2)
C3—C4—C5—C6	2.0 (3)	C10—N2—C9—N1	−2.1 (3)
C3—C4—C5—C7	−175.1 (2)	C9—N2—C10—O2	−174.9 (2)
C2—C1—C6—C5	0.1 (3)	C9—N2—C10—C11	3.9 (3)
C2—C1—C6—S1	−177.36 (17)	N1—C8—C11—C10	1.2 (3)
C4—C5—C6—C1	−1.8 (3)	S1—C8—C11—C10	179.05 (15)
C7—C5—C6—C1	175.2 (2)	N1—C8—C11—C12	177.94 (19)
C4—C5—C6—S1	175.71 (17)	S1—C8—C11—C12	−4.2 (3)
C7—C5—C6—S1	−7.3 (3)	O2—C10—C11—C8	175.5 (2)
C8—S1—C6—C1	−44.05 (19)	N2—C10—C11—C8	−3.3 (3)
C8—S1—C6—C5	138.44 (18)	O2—C10—C11—C12	−1.5 (3)
C9—N1—C8—C11	0.8 (3)	N2—C10—C11—C12	179.76 (18)
C9—N1—C8—S1	−177.26 (16)	C8—C11—C12—C13	−90.1 (2)
C6—S1—C8—C11	122.99 (18)	C10—C11—C12—C13	86.6 (2)
C6—S1—C8—N1	−59.01 (17)	C11—C12—C13—C14	177.06 (19)

### Hydrogen-bond geometry (Å, º)

Cg1 and Cg2 are the centroids of C1–C6 and C8–C11/N1/N2 rings, respectively.

**Table:**

*D*—H···*A*	*D*—H	H···*A*	*D*···*A*	*D*—H···*A*
C12—H12*B*···S1	0.97	2.75	3.166 (2)	107
N2—H1*N*2···O2^i^	0.82 (2)	2.01 (2)	2.829 (2)	171 (2)
N1—H1*N*1···O1^ii^	0.83 (3)	1.98 (3)	2.805 (2)	173 (2)
C7—H7*B*···O1^ii^	0.96	2.58	3.289 (3)	131
C2—H2*A*···*Cg*2^iii^	0.93	2.91	3.700 (2)	144
C7—H7*B*···*Cg*1^iv^	0.96	2.85	3.632 (3)	140

Symmetry codes: (i) −*x*+1, −*y*+1, −*z*; (ii) −*x*, −*y*+1, −*z*; (iii) −*x*, *y*+1/2, −*z*+1/2; (iv) −*x*, −*y*, −*z*.
